# Randomized Clinical Investigation of Titanium Implants with and without Platform Switching: Six Months’ Radiographic and Clinical Outcome

**DOI:** 10.3390/dj3020055

**Published:** 2015-04-23

**Authors:** Roberto Rossi, Diego Capri, Emanuele Risciotti, Paul Zeman

**Affiliations:** 1 Studio Dentistico, Torre San Vincenzo 2, Genova 16121, Italy; 2 Cobe Dental, Via Bazzanese 32/4, Casalecchio di Reno, Bologna 40033, Italy; E-Mail: cobedental@gmail.com; 3 Odontoiatria, Protesista Dentale, Corso EUROPA, 10, Milano 20122, Italy; E-Mail: studiorisciotti@tiscali.it; 4 Thommen Medical AG, Neckarsulmstrasse 28, Grenchen CH-2540, Switzerland; E-Mail: paul.zeman@thommenmedical.com

**Keywords:** dental implants, randomized clinical trial, platform shifting, marginal bone level

## Abstract

The aim of this study was to obtain a randomized, clinical and radiological comparison of implants with and without platform switching (PFS). The two compared titanium implant types differed only in the microgap position: test (PFS) or control (StE, no PFS). All implants were inserted in posterior regions and followed up for six months after abutment connection (AC). Twenty one patients with 21 PFS and 18 StE implants completed the six-month evaluation. No implant failed. One complication (exposed cap screw) was reported at AC. No statistically significant difference was observed between the marginal bone level of PFS and StE implants. Their bone level stabilized approximately 1 mm below the microgap. Based on the outcome of this short-term study with a limited number of patients, it seems unlikely that the optimal clinical and radiological outcome obtained with the tested standard implant (no PFS) can be improved by introducing an inward microgap shift (PFS).

## 1. Introduction

Replacement of missing teeth by endosseous titanium implants is currently a well-proven, routine procedure. Most importantly, it is a predictable and successful treatment option. With current implant systems, 10-year survival and success rates >90% have been achieved, e.g. [1]. Implant success is related to the maintenance of the crestal bone, a pre-requisite for implant stability and function. The formation of the biologic soft tissue coverage and the location of the implant-abutment junction in relation to the inevitable microgap pertinent to two-piece implants have been implicated as key factors in peri-implant bone remodelling [1].

In 2006, Lazzara and Porter [2] observed that placement of smaller prosthetic components on the implant platform, *i.e.*, shifting the implant abutment junction inwards, led to a shift of the inflammatory cell infiltrate closer to the central implant axis and away from the adjacent crestal bone. Since 2006, several implant manufacturers incorporated into their product lines a horizontal inward step at the implant abutment interface, *i.e.*, platform switching (PFS). This should enable a horizontal extension of the biological width. The number of papers dealing with PFS has increased over time [3]. It is nevertheless still undisputed that the finding of the beneficial effect of the inward microgap shifting was made serendipitously, and the scientific evidence is equivocal. Atieh *et al.* [4] performed a systematic review and meta-analysis of the influence of PFS around dental implants onto marginal bone preservation. Altogether, they included 10 controlled clinical trials with 1,239 implants in their analysis. The follow-up time varied between 12 and 60 months. Only one study showed a trend towards improved bone level preservation, and four studies showed no difference between PFS and platform matched implants. The remaining five trials demonstrated the contrary: in terms of marginal bone level preservation, platform-matched implants were significantly superior. The meta-analysis implied significantly less marginal bone loss around PFS as compared to platform-matched implants, but this effect was not accompanied by a significant difference in implant failures. It is also to be noted that the range of marginal bone loss varied to a large extent (PFS: 0.055–0.99 mm, platform-matched 0.19–1.67 mm). From the subgroup analysis, it appeared that only a PFS of at least 0.4 mm was associated with a more favorable bone response. The authors concluded therefore that PFS “may” preserve interimplant bone height and soft tissue levels. Marginal bone resorption seemed to be inversely related to the extent of the implant-abutment mismatch. A similarly cautious conclusion was drawn by Annibali *et al.* [5]. In their systematic analysis involving 435 patients and 993 implants, less bone loss was found with PFS implants, particularly with larger mismatching. The authors noted significant heterogeneity and possible publication bias of the reviewed results.

One of most recent reviews concluded that: clinical outcomes evaluated in human studies have not clearly shown the beneficial effect of the (PFS) concept. So far, no negative effects of PFS have been published. There are some concerns about situations with a small volume of soft tissue thickness [6].

Despite the extensive systematic analysis, randomized controlled studies are not yet available that would confirm the beneficial effects of the PFS concept [4,5,7]. A long-term prospective study has been published that was not included in the meta-analysis [4], as no control implants have been included in the longest known report on PFS implants [8]. Eighty percent of implants have lost 0.8 mm or less of marginal bone. A short-term (one year) randomized study was also published in parallel with the mentioned review [4] that did not confirm the hypothesis of a reduced peri-implant bone loss at implants restored according to the concept of platform switching [8].

Three different sizes of mismatch (inward shift of 0.5, 1.0 and 1.7 mm) were tested clinically and followed up for 33 months [9]. In this study, an inverse correlation was observed between the extent of platform “shift” and marginal alveolar bone loss. A similar observation was made in 10 patients that received 15 implants (implants with a body diameter of 5.0 mm were connected to 4.1-mm healing abutments) and with an 18 month follow-up time [10,11]. In the most recent dog study, one-piece, immediately-placed implants with a reduced diameter in their coronal aspect contributed to the preservation of the buccal crest compared with their non-reduced counterparts [12]. This finding supports the validity of the PFS concept.

The implant type tested in the presented study was selected as a state-of-the-art, cylindrical shape titanium implant with a micro-structured enossal surface and self-cutting threads. Its favorable five-year clinical and radiological outcomes have been demonstrated ecently [13,14,15,16]. The related three-year clinical outcome, e.g., with flapless placement and early or delayed loading, was also quite satisfactory [14]. No implant has failed after the three years follow-up. A very good clinical result was also obtained after a two-year clinical follow-up of 332 patients with 696 ELEMENT implants in a general practice setting. The observed overall survival rate was 99.6% [15]. The aim of the presented randomized clinical trial was therefore to test if the clinical outcome and crestal bone loss related to this specific implant type can be improved by introducing the PFS feature.

## 2. Materials and Methods

Three investigators, members of the “Boston University Italian Alumni” (BUIA) group, participated in this prospective, randomized clinical trial. They were skilled users of the tested implant system (ELEMENT RC, Thommen Medical AG, Grenchen, Switzerland). The clinical investigation was conducted in agreement with the principles set out by the Declaration of Helsinki (2008). All patients have provided written informed consent before any trial-related intervention. Both implant types have been CE certified and used within their approved indication range. The patients were therefore not subject to any additional risk, even if randomization was part of the study design, *i.e.*, the study-related interventions were not deemed to represent research. An ethical review board approval was therefore not sought for.

The trial was conducted as a pilot study. The sample size was selected empirically with no prospective sample size calculation. Patient selection criteria were defined as broadly as possible to ensure that the included patient population was representative of the general patient population. Those recruited were partially edentulous patients with at least one missing premolar or molar tooth (no incisors, no canines) in the mandible or the maxilla. The alveolus had to be healed, *i.e.*, tooth extraction performed at least three months before implant insertion. The insertion site had to have adequate bone horizontal volume (at least 7 mm) and acceptable mouth hygiene. Moderate smokers (less than 10 cigarettes per day or equivalent) and patients with a history of periodontitis were included. Patients were randomized to receive either the PFS (test) or the StE (control; both manufactured by Thommen Medical AG, Grenchen, Switzerland) implants, using a predefined randomization list. The surgeons were blinded to the inserted implant type until after patient screening, *i.e.*, the randomization code was revealed only after successful screening. If a patient needed more than one implant, the implant type not determined by randomization was inserted at the additional site(s).

Both PFS and StE implants were available with a platform diameter 5.0 mm and three different lengths (8.0, 9.5 and 11.0 mm). The PFS implants had the same design as the (commercially available) StE implants. They differed only in two aspects:

- the implant shoulder was beveled by 30 degrees to avoid a sharp edge that could lead to gingiva injury;
- a smaller diameter healing cap (4.0 mm) and abutment (EASY 4.0 mm; Thommen Medical AG, Grenchen, Switzerland) were used to ensure an inward microgap shift by 0.5 mm.

Both implant lines were EC certified and used within their approved indication range. Owing to the identical enossal geometry, both implant types were inserted with the same two-stage (submerged) surgical procedure, using the same surgical instruments, *etc.* The implants were set approximately 0.5 mm subcrestally, *i.e.*, with the machined-structured border 0.5 mm below the bone crest. The bone profiler was used, as needed.

Implants were inserted into healed alveoli at least 3 months after tooth loss, extraction or sinus lift. Patients with sinus lift were eligible if the implant healing was affected in the apical region only (no coronal bone augmentation). Patients that received bone replacement material at the implant site were excluded.

Eligible patients had to have adequate horizontal bone width, *i.e.,* a minimum of 7 mm, to ensure at least 1 mm of bone was available around the implant to enable placement of an implant with PF Ø5.0 mm, as well as quantitative assessment of bone level changes from X-rays. Care was taken to adhere with the parallel/perpendicular technique, so as to ensure constant projection over the study period. The three investigators (RR, DC, ER) therefore used customized jig bite blocks, which were connected to the ring film holder. Standard X-ray films were used that were digitalized (scanned), and the mesial and distal distance from implant shoulder to first bone-to-implant contact was measured (ImageJ, NIH, Version 1.49 m) [16]. The measured mesial and distal values were pooled for analysis.

**Figure 1:**
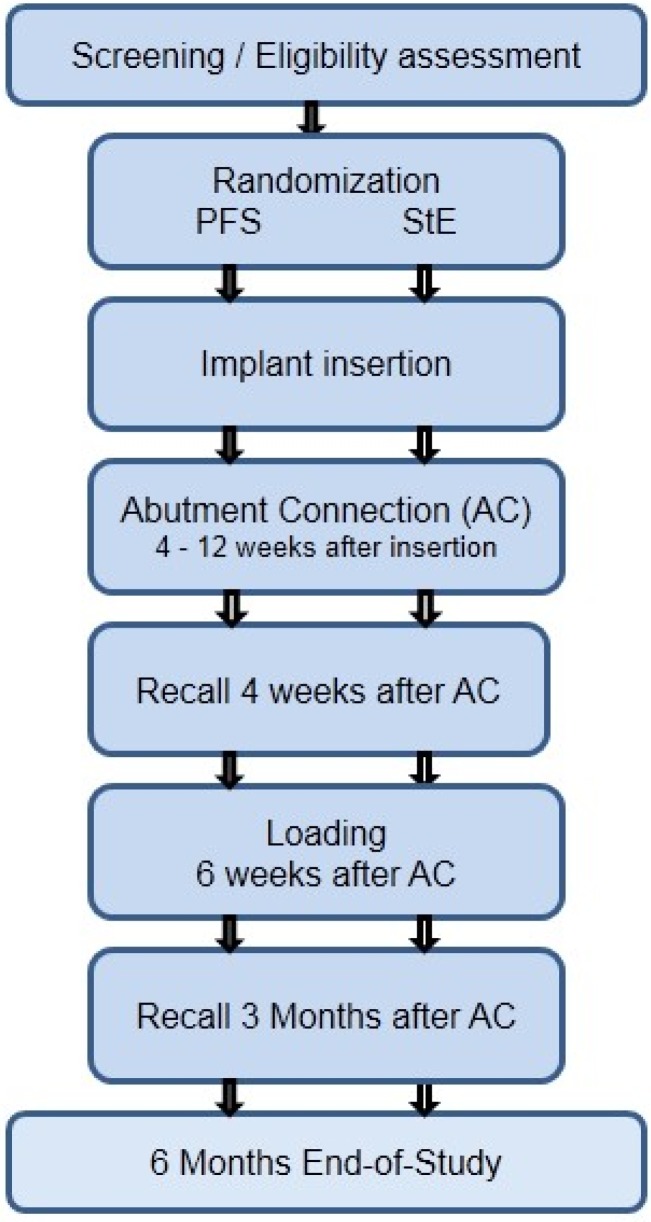
Study flow chart: overview of assessments. PFS, platform switching.

Second-stage surgery (abutment connection (AC)) was scheduled after a healing period of 4–12 weeks. Patients were recalled 4 weeks after AC, and a standard X-ray was obtained. Functional loading was done 6 weeks after AC when the permanent crown or a short-span (max 3 units) bridge had been screw-attached. Patients were followed up 3 and 6 months after AC. At both visits, a standard X-ray picture was taken, *i.e.*, five periapical X-rays were required during the study period of 6 months. Using a calibrated probe, parodontal status (Pocket Depth, PD and Attachment Height, AH) was evaluated at AC, at 6 weeks (occlusal loading) and 3 and 6 months after AC (see the flow diagram in Figure 1).

The patient data have been collected, and descriptive statistics has been calculated using commercially available software (Excel 2013). Statistical testing was performed for the quantitative (averaged mesial and distal) bone-to-implant distance. Parametric (*t*-tests) and non-parametric (Wilcoxon two-sample) statistical tests were calculated (SAS 9.3.1).

## 3. Results

Twenty three patients have been included in the study between January, 2010, and March, 2012. The mean patient age at implant insertion was 57 (35–78) years. They have been randomized to receive 21 PFS and 18 StE implants. Two patients have been screened and randomized, but were not able to undergo surgery. They were discontinued from the study, *i.e.*, 21 patients have completed the six months’ follow-up. All implants have been placed in posterior regions (x4–x7; FDI); 12 (31%) in the maxilla and 27 (69%) in the mandible, respectively. At the time of implant insertion, 65% of the patients had good or acceptable mouth hygiene. The implants have been inserted into healed bone (>3 months after tooth extraction). At 10 implant sites (six PFS, four StE), a previous bone augmentation (in the non-coronal region) has been done. For 13 implants (eight PFS and five StE), the maximal insertion torque of 30–45 Ncm was recorded, indicating good bone quality at the implant site.

No implant was lost during the trial period. All tested implants have been *in situ* and stable in the six months’ follow-up period. At the recall visit four weeks after the implant-abutment connection, a female patient with a PFS implant reported pregnancy. Further X-rays were therefore not taken for this patient. For another patient with a PFS implant, a partially exposed cover screw was reported also at the four weeks’ recall visit. There were no signs of infection. After gingiva adaptation, the site healed uneventfully.

**Figure 2:**
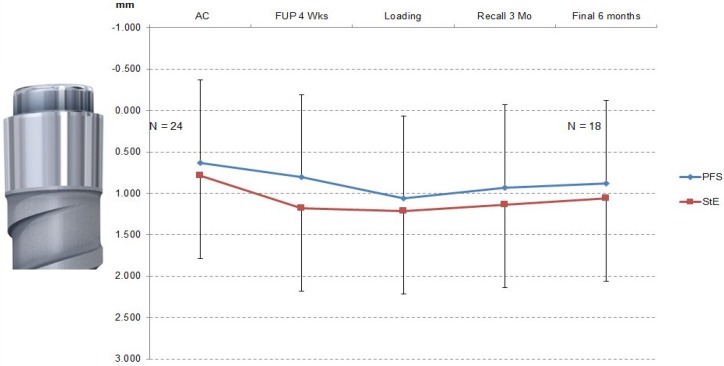
Bone level changes (error bars: PFS mean + standard deviation; StE mean: standard deviation). The first bone-to-implant contact was observed approximately 1 mm below the machined implant shoulder for both PFS and STE implants. The measurements were done on individual X-ray pictures (ImageJ). The implant abutment connection was used as the 0-mm level. The inserted picture of the machined implant shoulder corresponds to this level. Image scaling was based on the implant thread height (1 mm). The difference between PFS and StE implants was not statistically significant (Wilcoxon two-sample test).

The results of the quantitative X-ray evaluation are shown in Table 1 and Figure 2.

**Table 1:** Quantitative X-ray analysis. Results of the bone-to-implant distance measurement. Mesial and distal values were averaged.

Bone level (mm)	AC	FUP 4 weeks	Restoration 6 weeks	Recall 3 months	End-of-study 6 months
PFS					
Mean	0.63	0.81	1.06	0.93	0.88
SD	0.68	0.50	0.61	0.69	0.76
StE					
Mean	0.79	1.18	1.22	1.14	1.06
SD	0.48	0.39	0.62	0.63	0.51

Notes: AC, abutment connection; FUP, follow-up.

**Figure 3:**
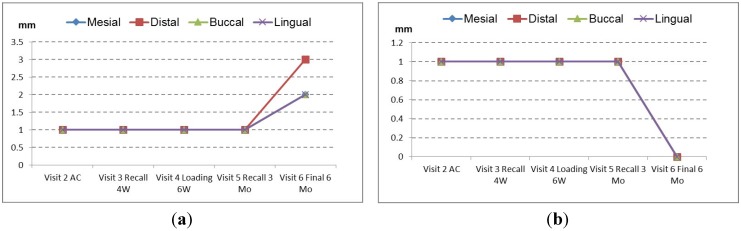
Changes of parodontal parameters. No difference was observed between PFS and StE implants. (**a**) Pocket depth; (**b**) attachment height.

The mesial and distal bone levels stabilized below the machined collar at the border of the machined and structured implant surface (machined collar height 1 mm). There was no statistically significant difference between the PFS and StE implants (Wilcoxon two-sample test), albeit the average bone loss with PFS implants appeared to be less at each time point. A similar picture was provided by the measurement of clinical parameters. Over the study period, the pocket depth (PD) and attachment height (AH) changes were similar for PFS and StE implants (Figure 3a,b, respectively).

**Figure 4:**
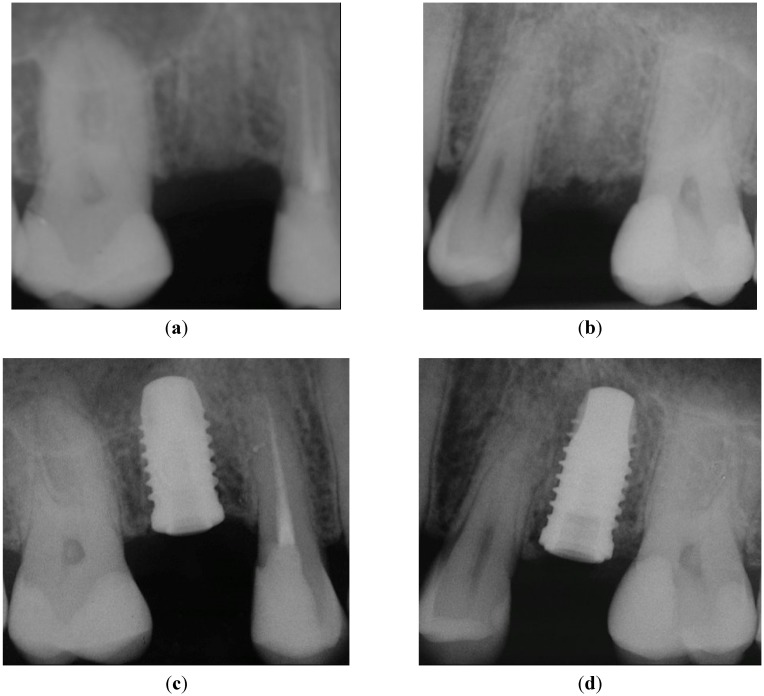
X-rays taken before implant insertion and immediately after implant placement: (**a**) healed crestal bone in Position 15; (**b**) healed crestal bone in Position 25. No pathological signs were present after implant placement of both implants: (**c**) Position 15, implant with PFS; (**d**) Position 25, StE implant.

**Figure 5:**
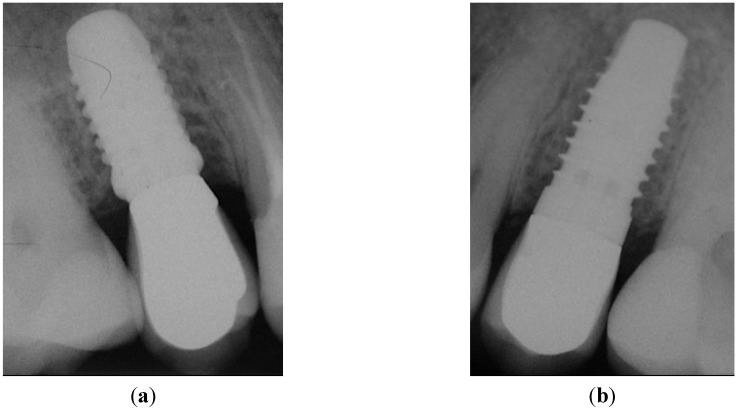
Peri-apical X-rays taken after AC. (**a**) Position 15; (**b**) Position 25. No pathological signs were visible around both implants.

**Figure 6:**
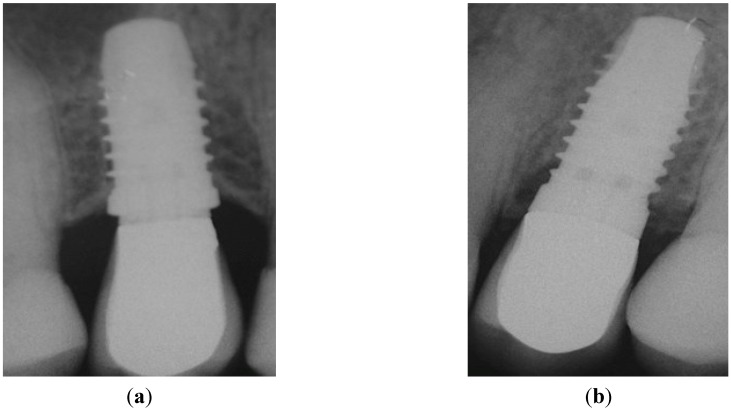
Peri-apical X-rays at one-year follow-up. (**a**) Position 15; (**b**) Position 25.

**Figure 7:**
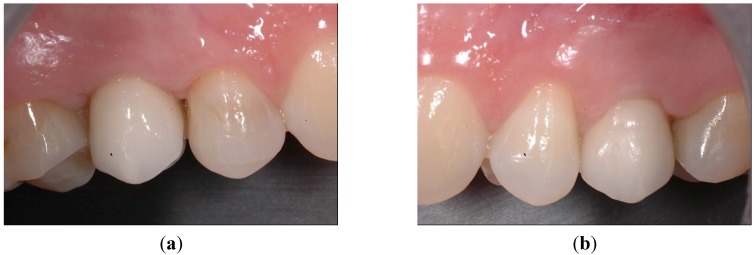

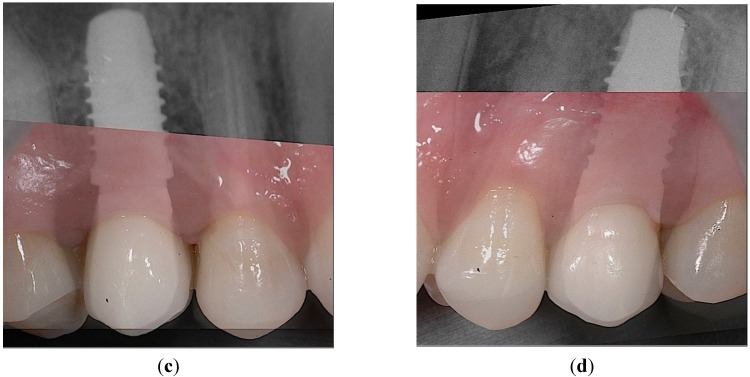
Intraoral views at one-year follow-up. (**a**) The optimal gingival margin was formed beneath the permanent crowns, both in the case of PFS, (**b**) as well as the StE implants. Superimposed clinical and X-ray pictures: (**c**) Position 15, PFS; (**d**) Position 25, StE.

This was particularly true in the first three months after AC (Visit 5). At the six months’ (end-of-study) visit, some increase in PD and AH loss was observed. The changes indicated healthy tissues around the implants, e.g., PD <4 mm. It also seems that attachment loss was somewhat less around StE implants. The comparable clinical outcome achieved by both PFS and StE implants is documented by a case of a single, “split-mouth” patient, which was treated within the presented study (Figure 4, Figure 5, Figure 6 and Figure 7).

## 4. Discussion

Presented in this report are the results of a prospective, randomized clinical study with a six months’ follow-up. No implant was lost, and the number of clinical complications was low (one cover screw exposure). This is also supported by the favorable outcome of the PD and AH measurement. In the trial period, all PD were <4 mm. The presented results do not contradict in any way the expected good clinical performance of the tested PFS and StE implants. This is in line with the published findings that implants with PFS may by beneficial. As confirmed also by the most recent review, evidence from long-term, randomized clinical trials are nevertheless still lacking [7].

The loss of the marginal bone was the same for the PFS and StE implants. As shown in Table 1 and Figure 2, the mean mesial and distal bone level at AC was 0.63 mm for PFS and 0.79 mm for StE implants. The starting values document that both implant types have been inserted into the same depth. The distance between implant shoulder and first bone-to-implant contact did not change six months later, *i.e.*, it was 0.88 mm and 1.06 mm for PFS and StE implants, respectively. There seems to be nevertheless a tendency towards less marginal bone loss with the PFS implants. A longer follow-up time, as well as a larger number of patients and implants could help to confirm this apparent trend, in agreement with the most recent meta-analysis that showed a significant marginal bone difference in favor of PFS implants [17].

For both the PFS and StE implants, the bone level has stabilized at the level that was described previously. The experience of most of the users of the tested implant type, that in the long term, the crestal bone stabilizes 1 mm below the IAC, *i.e.*, at or above the level of the first implant thread [13,14,15,16,18], was clearly confirmed by this trial. This finding seems to be true for currently available implants with moderately rough enossal surfaces [19]. The question therefore arises whether the predictable bone loss, *i.e.*, its stabilization just beneath the machined collar after five years’ follow-up, can be improved by PFS.

While some crestal bone loss of 1 to 2 mm in the initial phase after placement and during the first year of loading has been observed and described as a normal effect of the bone remodeling process after a surgical trauma, continuous progression of bone loss should not occur or not exceed 0.2 mm per year [17]. In this study, the radiological observation was started with AC. Six months later, the marginal bone level has stabilized 1 mm below the implant shoulder, *i.e.,* at the level of the first implant thread. Only minimal (<0.1 mm/year) further marginal bone loss occurred in this study. This was confirmed also in two separate studies that comprised a five years’ follow-up [13,17]. This finding is not supported by the outcome of the most recent comparison of platform-switched and platform matching implants. In this meta-analysis, twenty-eight publications were included with a total of 1216 platform-switched and 1157 platform-matched implants. Significantly less marginal bone loss was observed with PFS than with platform matching implants. The difference of marginal bone loss increased significantly (*p* < 0.001) with both longer follow-up time and increasing mismatch between implant platform and the abutment [17]. The authors noted that the results should be interpreted with caution due to presence of confounding factors in the included studies, most of them with short follow-up periods. Randomized clinical trials with a sufficient number of patients and long-term follow-up are still lacking.

The same question applies to soft tissue changes. In the presented study, the implants have been placed by experienced surgeons that achieved a very favorable clinical outcome (PD < 4 mm, Figure 3a). It seems unlikely that such an optimal outcome can be further improved significantly by PFS. The documentation of a case from the presented collection is shown below to support this notion. A 35-year-old female patient required the replacement of the second premolar on both maxillary arches (Positions 15 and 25; FDI). Minimally traumatic tooth extraction was done eight months before implantation; consequently, the crestal bone immediately before implant placement was fully healed (Figure 4a and Figure 4b, Positions 15 and 25, respectively). Implants were placed with no complications. Following randomization, the PFS implant was inserted on the left (Figure 4c; Position 15), whereas StE was placed on the right side (Figure 4d; Position 25).

The implants have been functionally loaded (AC) after six weeks. Corresponding peri-apical X-rays taken at this visit confirmed the absence of any pathological signs (Figure 5a,b; Positions 15 and 25, respectively).

Six months after study completion (at the one-year follow-up), osseointegration was checked radiographically. Both X-rays were free of any pathological signs (Figure 6a,b) with a quite satisfactory clinical outcome (Figure 7a–d). The outcome of this “split-jaw” case was equally positive for both the PFS (Position 15) and StE (Position 25) implants. In this respect, the case is representative of the entire trial patient population.

## 5. Conclusions

Based on the outcome of this small sample size, prospective, randomized clinical trial with six months’ follow-up, a positive effect of PFS on marginal bone level changes or on clinical outcome was not confirmed. No significant differences have been found, neither in the radiologic (quantitative crestal bone level changes), parodontal (pocket depth, attachment height) or clinical outcomes between implants with (PFS) or without (StE) platform switching. A large-scale, long-term clinical trial would be needed to confirm, or reject, the hypothesis of PFS superiority. With the titanium implants used in this study, very good outcomes were obtained after five years. It seems unlikely that these outcomes can be improved by PFS in a clinically meaningful way, *i.e.*, that the optimal clinical and radiological outcome obtained with implants without PFS can be further improved by introducing an inward microgap shift (PFS).
